# Enrichment of *Fusobacteria* in Sea Surface Oil Slicks from the Deepwater Horizon Oil Spill

**DOI:** 10.3390/microorganisms4030024

**Published:** 2016-07-27

**Authors:** Tony Gutierrez, David Berry, Andreas Teske, Michael D. Aitken

**Affiliations:** 1 School of Life Sciences, Heriot Watt University, Edinburgh, EH14 4AS, UK; 2 Department of Environmental Sciences and Engineering, Gillings School of Global Public Health, University of North Carolina at Chapel Hill, Chapel Hill, NC 27599, USA; mike_aitken@unc.edu; 3 Department of Microbiology and Ecosystem Science, Division of Microbial Ecology, University of Vienna, A-1090 Vienna, Austria; berry@microbial-ecology.net; 4 Department of Marine Sciences, University of North Carolina at Chapel Hill, Chapel Hill, NC 27599, USA; teske@email.unc.edu

**Keywords:** biodegradation, *Deepwater Horizon* oil spill, Gulf of Mexico, marine bacteria, *Fusobacteria*, obligate anaerobes, marine oil snow, pyrosequencing

## Abstract

The Deepwater Horizon (DWH) oil spill led to rapid microbial community shifts in the Gulf of Mexico, including the formation of unprecedented quantities of marine oil snow (MOS) and of a massive subsurface oil plume. The major taxa that bloomed in sea surface oil slicks during the spill included *Cycloclasticus*, and to a lesser extent *Halomonas*, *Alteromonas,* and *Pseudoalteromonas*—organisms that grow and degrade oil hydrocarbons aerobically. Here, we show that sea surface oil slicks at DWH contained obligate and facultative anaerobic taxa, including members of the obligate anaerobic phylum *Fusobacteria* that are commonly found in marine sediment environments. Pyrosequencing analysis revealed that *Fusobacteria* were strongly selected for when sea surface oil slicks were allowed to develop anaerobically. These organisms have been found in oil-contaminated sediments in the Gulf of Mexico, in deep marine oil reservoirs, and other oil-contaminated sites, suggesting they have putative hydrocarbon-degrading qualities. The occurrence and strong selection for *Fusobacteria* in a lab-based incubation of a sea surface oil slick sample collected during the spill suggests that these organisms may have become enriched in anaerobic zones of suspended particulates, such as MOS. Whilst the formation and rapid sinking of MOS is recognised as an important mechanism by which a proportion of the Macondo oil had been transported to the sea floor, its role in potentially transporting microorganisms, including oil-degraders, from the upper reaches of the water column to the seafloor should be considered. The presence of *Fusobacteria* on the sea surface—a highly oxygenated environment—is intriguing, and may be explained by the vertical upsurge of oil that provided a carrier to transport these organisms from anaerobic/micro-aerophilic zones in the oil plume or seabed to the upper reaches of the water column. We also propose that the formation of rapidly-sinking MOS may have re-transported these, and other microbial taxa, to the sediment in the Gulf of Mexico.

## 1. Introduction

The Deepwater Horizon (DWH) oil spill, recorded as the largest oil spill in US waters, commenced on 20th April of 2010 and lasted for about 84 days until 15 July when the leaky Macondo-252 oil well was finally capped. The spill resulted in the release of about 4.1 million barrels (ca. 651 million litres) of crude oil into the Gulf of Mexico from a depth of 1500 m, and the application of about 2.1 million gallons (ca. 7.9 million litres) of dispersants (Corexit EC9500A and EC9527A) directly at the leaky wellhead and on sea surface oil slicks [1,2,3]. A distinctive feature that set the DWH spill apart from other oil spills at sea was the formation of a hydrocarbon-enriched plume that became entrained at 1000–1300 m within the water column and that triggered dramatic microbial shifts, notably of members belonging to the *Oceanospirillales* (termed DWH *Oceanospirillales*), *Cycloclasticus*, and *Colwellia*, which were significantly enriched within the plume [4,5,6,7,8]. Oil from the leaky Macondo-252 well also reached the sea surface in the Gulf of Mexico, forming expansive oil slicks that also triggered dramatic microbial community shifts—notably an enrichment of *Cycloclasticus* and, to a lesser extent, *Halomonas*, *Alteromonas,* and *Pseudoalteromonas* [8,9]. These oil-degrading bacteria play a fundamental role in the oxidation and biodegradation of oil hydrocarbons in the marine environment, and their important role in the removal of the Macondo oil in sea surface slicks and the subsurface plume at DWH is unquestionable [10,11].

Of the total amount of oil that was released from the leaky Macondo well, approximately 60% was estimated to have reached the sea surface [12], where it became exposed to various physical (wind/current-driven forcing) and chemical (evaporation, photo-oxidation) weathering processes that altered the composition of the oil [13], including the formation of water-in-oil emulsions [14]. A distinctive feature of the DWH spill was the formation of marine oil snow (MOS; *aka* oil aggregates). MOS of macroscopic cm-size dimensions can be described as a mucilaginous floating organic matter with a “fluffy” or gelatinous off-white appearance, containing oil droplets embedded within its amorphous matrix. MOS was observed during the first research cruise on *R/V Pelican* to the DWH site in early May 2010, and was frequently encountered around the vicinity of surface oil slicks and in the subsurface oil plume [14,15]. Here we investigated the occurrence and enrichment of anaerobic bacteria, with putative oil-degrading abilities, in sea surface oil slicks at DHW. The sea surface is, in general, a highly oxygenated zone, so the identification of anaerobic taxa in these waters, and their enrichment by the Macondo oil, is intriguing. Anaerobic micro-niches that could support a community of obligate and/or facultative anaerobic organisms can exist in oxygenated waters of the sea surface. For example, studies using oxygen microelectrodes to measure dissolved oxygen in cyanobacterial colonies, zooplankton faecal pellets, and planktonic/detritus aggregates have revealed that even the tiniest of marine snow particles can contain anaerobic niches [16,17,18,19,20] and could support the growth of obligate anaerobic or microaerophilic microorganisms in these ephemeral habitats [21]. The creation of anaerobic niches inside MOS particles could occur when microbial respiration rates inside the particles are high enough to utilise the available oxygen faster than it can be replenished from the surrounding seawater environment. The formation of an oxygen gradient, which is increasingly more anoxic toward the interior of the particles, would have a controlling influence on the stratification of the microbial community. Essentially, this could lead to enriching for obligate and/or facultative anaerobes and their colonization within the interior anaerobic niches of the MOS particles.

In this study we investigate the formation of the bacterial community associated with sea surface oil slicks during the early phase of the DWH oil spill, herein with the focus to identify anaerobic oil-degrading taxa. For this, a surface oil slick water sample collected during the first two weeks of the DWH blowout was incubated in a hermetically-sealed environment so that oxygen levels could gradually decrease, in order to assess the possible enrichment of anaerobic oil-degrading bacteria. So far, sequencing surveys performed on Gulf of Mexico water column samples collected during the DWH spill had not detected obligately anaerobic taxa. Our objective was to test the hypothesis that anaerobes with putative oil-degrading capabilities were present in sea surface waters of the Gulf during the spill. We hypothesise that these organisms persisted in surface oil slicks because they occupied anaerobic micro-niches in the water column such as the interior of MOS particles, and their strong enrichment suggests they contributed to the overall biodegradation of the oil in the Gulf. The presence of these organisms in surface waters also raises interesting questions to their genesis and fate during the spill, and we explore some possible reasons to explain this.

## 2. Materials and Methods

### 2.1. Field Sample Collection and Incubation

During a research cruise on the *R/V Pelican* on 5th May of 2010 (i.e., 15 days after the DWH blowout), oil-contaminated water samples were collected from the sea surface at 0.86 m from the site of the DWH spill (28°44.175′ N, 88°22.335′ W) in the Gulf of Mexico. At the time of collection, fractions of the samples were filtered onto 0.2-μm pore-size Nucleopore filters (Whatman, Maidstone, UK) and stored frozen for DNA extraction and barcoded 16S rRNA gene pyrosequencing, the results of which are described in detail elsewhere as the PE5 bacterial community of sea surface oil slicks [8]. A sub-sample (250 mL) of this PE5 sea surface oil slick sample was incubated at 21 °C in the dark with gentle shaking in an hermetically-sealed 250-mL wide-neck glass bottle, with a low headspace volume (ca. 100 cm^3^) above the water surface in order for the sample to become anaerobic over time. The sample had a visible layer of Macondo oil floating at the air–water interface—this oil formed part of the original PE5 water sample at the time of collection in the Gulf. After 4 weeks the sample incubation was manually shaken, and then 20 mL of the incubation was filtered onto a 0.2-μm pore-size Nucleopore filter and stored frozen for DNA extraction and barcoded amplicon pyrosequencing—referred to hereafter as the TG1 water sample.

### 2.2. Barcoded Amplicon Pyrosequencing

DNA was extracted from the TG1 frozen sample using the method of Tillett and Neilan [22], and barcoded 16S rRNA gene pyrosequencing was performed to analyse the bacterial community of the sample. Ten-fold dilutions of extracted DNA in water were used as template for triplicate PCR reactions for each sample. Primer pairs 27f and 338r were modified to incorporate an identical eight-base-pair (bp) barcode sequence unique to each sample and a 2-bp spacer on the 5′ end of the primer sequence [23]. Each 20 µL PCR reaction was run for 25 cycles at 94 °C for 45 s, 55 °C for 45 s, and 72 °C for 1 min on an Eppendorf (Eppendorf, Westbury, NY, USA) Mastercycler Gradient thermal cycler before verification of the proper amplicon size on a 1% agarose gel. The triplicate reactions for each sample were pooled and purified with a QIAquick PCR Purification Kit (Qiagen, Valencia, CA, USA) and eluted in 30 µL of 10 mM Tris-Cl (pH 8.5) buffer. The DNA concentration of pooled amplicons was then measured using a NanoDrop ND-3300 Fluorospectrometer (Thermo, Waltham, MA, USA) and Quant-iT Picogreen dsDNA Kit (Invitrogen, Carlsbad, CA, USA) prior to combining into a single sample at a concentration suitable for pyrosequencing. The sample was submitted to the High-Throughput Sequencing Facility at the University of North Carolina-Chapel Hill for sequencing using the 454 Life Sciences Titanium platform (Roche Diagnostics, Branford, CT, USA).

Pyrosequencing reads were trimmed and filtered using the LUCY program with a minimum PHRED score of 27.5 and minimum length of 200 nt to remove low-quality regions and short reads [24]. Reads were de-multiplexed based on the 8 bp barcode sequence, and the primer and barcode regions were removed using QIIME [25]. To form operational taxonomic units, the reads were clustered at 97% sequence identity with UCLUST [26], and the most abundant unique read within each cluster was used as its representative sequence. Initial phylogenetic identification was made using BLAST [27], and chimeras were detected with Chimera Slayer [28]. Sequence data were submitted to the European Nucleotide Archive Sequence Read Archive under the study accession number ERP002443.

### 2.3. Phylogenetic Tree

Operational taxonomic units (OTUs) classified to the phylum *Fusobacteria* in this and related studies were extracted for comparison. OTUs were identified as enriched in the TG1 sample relative to the initial sea surface oil slick water sample (PE5) using two criteria: (1) an increase of ≥1% relative abundance; and (2) statistically-significant enrichment, as determined with a two-proportion *T*-test and correcting *P*-values for multiple comparisons using the false discovery rate method in *R* [29]. The 16S rRNA sequences of *Fusobacteria* were aligned and manually curated in ARB [30]. Closely-related full length sequences from the SILVA SSU NR 123 NR database [31] were used for tree construction using a maximum likelihood method (RAxML) with 100 bootstraps. *Halomonas neptunia* (AF212202) was used as an outgroup. Fusobacterial sequences were then added using the quick-add parsimony method in ARB.

## 3. Results and Discussion

**Figure 1:**
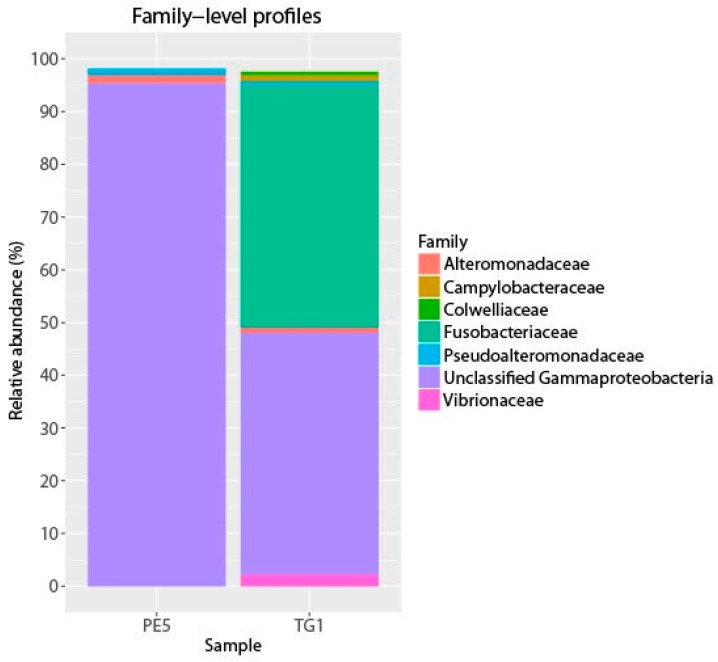
Abundant bacterial phyla in starting (PE5) and incubated (TG1) samples. Phyla with at least 0.5% relative abundance in either library are shown.

Barcoded 16S rRNA gene pyrosequencing was used to analyse and compare the bacterial community of DWH sea surface oil slicks (incubated to progressively develop anaerobic conditions; the TG1 water sample) to that of sea surface oil slicks collected 15 days after the DWH blowout (i.e., the PE5 water sample). As reported by Yang, et al. [8], the PE5 bacterial community structure was dominated by “specialist” polycyclic aromatic hydrocarbon (PAH)-degrading bacteria of the genus *Cycloclasticus*, and of hydrocarbon-degrading “generalists” that included members of the genera *Alteromonas*, *Colwellia*, *Pseudoalteromonas*, and, to a lesser extent, *Halomonas*. These organisms are members of the *Gammaproteobacteria* and they have been shown to participate in MOS formation, to produce exopolymeric substances (EPS), and in the particular case of *Halomonas*, its produced EPS was shown to enhance the bioavailability and subsequent biodegradation of aromatic hydrocarbons ([4,32]; T. Gutierrez, K. Salek, M. Jones, A.Teske, unpublished data). In Figure 1, the bacterial community structure—at family-level classification—of the TG1 water sample is presented alongside that of the PE5 sample. Of a total of 23,775 high quality partial gene sequences, the TG1 bacterial community structure shared similarities to that of the PE5 community, with a clear and persistent dominance of the *Cycloclasticus* (43% of total sequences; Table 1). *Cycloclasticus* are, however, strictly aerobic organisms, so their high abundance in the TG1 incubation is interesting. Most studies have reported the identification of *Cycloclasticus* in marine sediments from shallow and also deep waters [33,34,35,36,37,38] where oxygen concentrations can range from oxic to anoxic within the first couple of millimetres depth in the sediment. The dominance of *Cycloclasticus* in the TG1 incubation, therefore, may be explained by their inherent ability to tolerate microaerophilic/anaerobic conditions, allowing these organisms to have persisted in high abundance in this incubation—these organisms had already been strongly enriched in surface oil slicks at DWH, and thus dominated the microbial community from the commencement of the TG1 incubation. Alternatively, these organisms were in a dead, dying, or dormant phase after displaying a “boom” followed by “bust” population dynamics, as is typical for these heterotrophic copiotrophs. Even dead or dormant bacterial cells can still harbour intact DNA [39]. The community also comprised phyla within the *Bacteroidetes*, *Firmicutes,* and *Epsilonproteobacteria*, and a complex gammaproteobacterial assemblage that mainly included members of the order *Vibrionales* and *Congregibacter*—groups that include members inhabiting microaerophilic or anaerobic environments. The relative abundances of these organisms in the TG1 library is shown alongside that of the PE5 library in Table 1.

**Figure 2:**
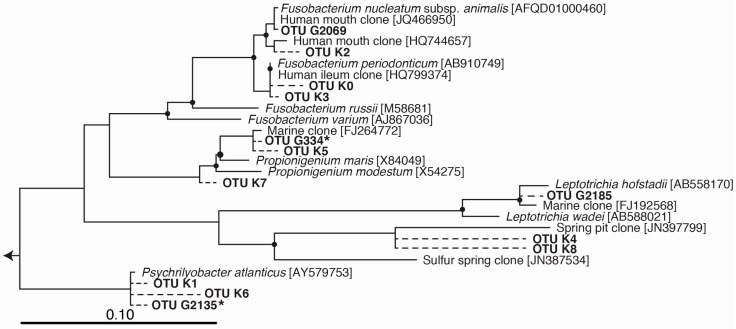
Phylogenetic tree of *Fusobacteria* operational taxonomic units (OTUs) enriched in incubations (marked with an asterisk) or detected in Deepwater Horizon surveys (OTUs beginning with “K” from Kleindienst, et al. [6], and OTUs beginning with “G” from Gutierrez, et al. [9]). OTU sequences were added to a bootstrapped RAxML tree of full-length sequences via quick-add parsimony without modifying tree topology to demonstrate the approximate phylogenetic placement of indicators (dashed lines). Nodes with bootstrap support of at least 80% are marked with a black circle. Bar indicates 10 substitutions per 100 nucleotide positions.

The most notable feature of the TG1 microbial community was the dramatic increase in members belonging to the *Fusobacteria* (46% of total sequences; Figure 1, Table 1)—a phylum of obligately anaerobic bacteria [40]. Two OTUs within the family *Fusobacteriaceae*—G334 and G2135—were markedly enriched, and together constituted 39.8% of the TG1 community. These *Fusobacteria* OTUs increased from undetectable levels in the PE5 community to respectively constituting 18.4% and 21.4% of the total community composition in the TG1 library. Sequencing surveys of surface oil slicks at DWH, however, had not identified these organisms, and to the best of our knowledge, *Fusobacteria* have rarely been detected in subsurface water column sequencing surveys of the Gulf of Mexico during and after the spill. In a study by Yang, et al. [8], *Fusobacteria* (represented by OTU 2069 in Figure 2) were identified in a water sample (designated B1) collected on 31 May 2010 during the active phase of the DWH spill at 1320 m depth (corresponding to just below the oil plume). The same study identified one other *Fusobacteria* (represented by OTU 2185 in Figure 2) from a water sample (designated GIP22) collected on 18 October 2010, well after capping of the leaky wellhead, from a depth of 1052 m; however, their relative abundances were <0.2%. In a recent paper by Kleindienst, et al. [6], *Fusobacteria* sequences were also identified in sequencing surveys of the Gulf of Mexico water column from samples obtained before (OTUs K1-K4 and K7), during (from above the oil plume; OTUs K0 and K1), and after the spill (from near the seafloor; OTUs K1, K5, K6, and K8); but again, at relative abundances of <0.2%. The *Fusobacteria* sequences from these studies are presented together with those identified enriched in the TG1 water sample (OTUs G334 and G2135) and with close relatives on the phylogenetic tree shown in Figure 2.

Collectively, *Cycloclasticus* and *Fusobacteria* constituted 89.2% relative abundance of the TG1 community (Table 1). Despite the rare occurrence of *Fusobacteria* in the Gulf of Mexico water column, these organisms have more frequently been encountered in coastal and off-shore sediment environments of the Gulf, in particular at sites impacted by the DWH oil [41,42,43]. *Fusobacteria* have been found in deep marine oil reservoirs (e.g., Wang, et al. [44]), and a representative *Fusobacteria* whole genome encoded the complete pathway for polycyclic aromatic hydrocarbon degradation [45]. These findings suggest a possible role for these organisms in the biodegradation of the Macondo oil during the DWH spill. The ability to degrade hydrocarbons has not yet been substantiated for any member of this phylum, and thus warrants investigation.

Based on our detection of *Fusobacteria* in surface oil slick samples collected during the DWH oil spill, and their strong enrichment in the presence of the Macondo oil (with respect to the TG1 incubation), we posit that these organisms may have become strongly enriched in sea surface waters during the spill, but only within anaerobic niches. The formation and copious presence of MOS in surface waters may have provided this niche. We hypothesize that the subsequent rapid-sinking of MOS could have played a role in seeding, or replenishing, newly-forming surficial sediment layers in the Gulf of Mexico with *Fusobacteria* and other MOS-associated taxa. Supporting this hypothesis, MOS formed in lab-based experiments using oil-contaminated sea surface water collected near DWH within two weeks of the spill were found to harbour *Cycloclasticus* as a dominant member of the MOS-associated bacterial community [46]. *Cycloclasticus* was also found in high abundance in freshly-deposited seafloor sediments in the Gulf following the DHW spill [47]. Collectively, this suggests a role for MOS in the vertical transport of sea surface microorganisms to the sediment.

The study by Arnosti, et al. [46] represents the only published report to-date that has examined the microbial communities associated with MOS. The authors showed that the MOS-associated bacterial community composition was distinctly different from the free-living (surrounding seawater) community. MOS-associated communities were found to be primarily composed of oil-degrading and exopolysaccharide-producing members of the *Gammaproteobacteria* (principally *Cycloclasticus*, *Congregibacter*, *Haliela*, *Halomonas*, *Marinobacter*), and diverse members of the *Alphaproteobacteria* (principally the *Roseobacter* clade), and some members within the *Bacteroidetes* and *Planctomycetales*. With the exception of the *Planctomycetales*, members of these other taxa have been reported with hydrocarbon degradation qualities. Members of all of these taxa are generally aerobic heterotrophs, with the exception of *Congregibacter*, which is a facultative anaerobic anoxygenic photosynthesizer (AAnP) and an oligotrophic specialist [48]. The detection of obligately anaerobic microorganisms may have been missed in MOS particles due to the shallow sequencing depth that is inherent to Sanger sequencing of clone libraries used in the survey by Arnosti, et al. [46]. The authors did not, however, identify any sequence of *Fusobacteria*, though this may be because these organisms comprised the fraction of rare taxa that could not be detected by the clone library approach employed in their study. Microbial respiration creates anoxic conditions, so that diverse aerobic and anaerobic microorganisms associated with MOS particles would be expected to colonise different niches within these particles. The occurrence of facultative anaerobic organisms (e.g., *Congregibacter*) in MOS particles is indicative that such particles may have developed internal anaerobic niches that supported the growth of these organisms. Low oxygen environments in the water column from where *Fusobacteria* could have been transported to the sea surface include the relatively oxygen-depleted deepwater plume [49], the Gulf of Mexico dead zone, and sediment.

Obligate anaerobes, such as the *Fusobacteria*, may be expected to maintain low abundance levels in MOS particles. However, it should be noted that low cell abundance for any particular microbial group in situ does not always equate to it being in a state of metabolic inactivity, and this may be the case for the *Fusobacteria*, as they were strongly enriched in the TG1 incubation. MOS particles formed during the DWH spill, which could harbour anaerobic oases and contain associated Macondo oil, could have therefore afforded a refuge and vehicle for the transport of these organisms to the Gulf of Mexico sediment. 

## 4. Conclusions

In summary, our findings demonstrate that obligate anaerobes—such as members of *Fusobacteria* with putative hydrocarbon-degrading qualities—were present in sea surface oil slicks during the Deepwater Horizon oil spill. We hypothesise that these organisms may have become enriched in anaerobic zones of suspended particulates, such as MOS, whereby they may have subsequently become transported from the sea surface to the sediment. The identification of obligately anaerobic ***Fusobacteria*** in the sea surface of the Gulf of Mexico is quite intriguing, given that this is a highly aerobic zone. Further work, however, will be needed to more fully unravel the role of these organisms in the event of oil contamination in surface waters.

## Figures and Tables

**Table 1:** Overview of phylogenetic affiliations of bacterial 16S rRNA gene sequences and their relative abundances in the PE5 sea surface oil slick collected 2 weeks after the DWH blowout [8] and the TG1 sea surface oil slick sample incubated for 4 weeks (this study).

Phylogenetic Affiliation	PE5 Incubation		TG1 Incubation	
	% Relative Abundance	No. of Sequences	% Relative Abundance	No. of Sequences
Alphaproteobacteria				
Oceanibaculum	0.00	0	0.00	1
Rhodospirillales	0.00	2	0.00	1
Roseobacter	0.06	28	0.01	3
SAR11 (candidatus Pelagibacter)	0.02	8	0.00	1
SAR11 (SAR407)	0.03	14	0.00	0
SAR11 (SAR464)	0.00	0	0.00	1
SAR11 (unnamed)	0.00	0	0.00	1
Unclassified	0.00	2	0.00	0
Gammaproteobacteria				
Acinetobacter	0.00	0	0.00	0
Agarivorans	0.00	2	0.03	15
AGG47 related	0.00	1	0.00	0
Alcanivorax	0.00	1	0.00	0
Alteromonadales	0.09	41	0.00	1
Alteromonas	0.73	349	0.01	3
Arctic 96BD19	0.00	0	0.00	1
Colwellia	0.09	41	0.18	84
Colwellia related	0.02	8	0.01	7
Congregibacter and relatives	0.03	13	0.46	219
Cycloclasticus	47.03	22,333	21.78	10,285
Cycloclasticus related	2.24	564	1.19	439
DWH plume group	0.04	9	0.02	6
Halomonas	0.15	36	0.00	0
Idiomarina	0.00	0	0.00	1
Marinimicrobium	0.00	0	0.00	1
Marinobacter	0.02	5	0.01	2
Marinomonas	0.00	0	0.05	19
Moritella	0.00	1	0.10	37
Neptunomonas	0.00	0	0.01	2
Oceanobacter	0.00	0	0.00	1
Oceanospirillales	0.03	7	0.03	10
Oceanospirillum	0.00	0	0.02	8
Oleispira	0.03	7	0.03	10
Pseudoalteromonas	1.17	287	0.71	260
SAR86	0.02	4	0.00	0
Shewanella	0.00	0	0.02	8
SUP05 Arctic related	0.00	1	0.00	1
Unidentified	0.05	13	0.39	142
Vibrionales	0.02	5	1.82	654
ZD0417	0.00	1	0.00	0
Bacteroidetes	0.43	104	0.64	227
Cyanobacteria	0.30	73	0.01	3
Deltaproteobacteria	0.05	12	0.03	9
Epsilonproteobacteria	0.04	10	0.75	264
Firmicutes	0.00	0	0.28	98
Fusobacteria	0.00	0	31.46	10,926
Actinobacteria	0.01	4	0.00	0
Lentisphaerae	0.00	0	0.08	20
Planctomycetes	0.02	4	0.00	0
Fibrobacteres/Acidobacteria	0.00	0	0.00	1
Firmicutes	0.02	5	0.00	0
Unclassified bacteria	0.02	4	0.01	3
**Total sequences**		**23,999**		**23,775**
